# Cellular Electrophysiology of Iron-Overloaded Cardiomyocytes

**DOI:** 10.3389/fphys.2018.01615

**Published:** 2018-11-15

**Authors:** Natthaphat Siri-Angkul, Lai-Hua Xie, Siriporn C. Chattipakorn, Nipon Chattipakorn

**Affiliations:** ^1^Cardiac Electrophysiology Research and Training Center, Faculty of Medicine, Chiang Mai University, Chiang Mai, Thailand; ^2^Center of Excellence in Cardiac Electrophysiology Research, Chiang Mai University, Chiang Mai, Thailand; ^3^Department of Cell Biology and Molecular Medicine, Rutgers University – New Jersey Medical School, Newark, NJ, United States; ^4^Cardiac Electrophysiology Unit, Department of Physiology, Faculty of Medicine, Chiang Mai University, Chiang Mai, Thailand; ^5^Department of Oral Biology and Diagnostic Sciences, Faculty of Dentistry, Chiang Mai University, Chiang Mai, Thailand

**Keywords:** cardiomyocyte, iron, ion channel, ionic current, electrophysiology

## Abstract

Iron, the most abundant transition metal element in the human body, plays an essential role in many physiological processes. However, without a physiologically active excretory pathway, iron is subject to strict homeostatic processes acting upon its absorption, storage, mobilization, and utilization. These intricate controls are perturbed in primary and secondary hemochromatoses, leading to a deposition of excess iron in multiple vital organs including the heart. Iron overload cardiomyopathy is the leading cause of mortality in patients with iron overload conditions. Apart from mechanical deterioration of the siderotic myocardium, arrhythmias reportedly contribute to a substantial portion of cardiac death associated with iron overload. Despite this significant impact, the cellular mechanisms of electrical disturbances in an iron-overloaded heart are still incompletely characterized. This review article focuses on cellular electrophysiological studies that directly investigate the effects of iron overload on the function of cardiac ion channels, including trans-sarcolemmal and sarcoplasmic reticulum Ca^2+^ fluxes, as well as cardiac action potential morphology. Our ultimate aim is to provide a comprehensive summary of the currently available information that will encourage and facilitate further mechanistic elucidation of iron-induced pathoelectrophysiological changes in the heart.

## Introduction

Iron is an essential metal element that serves many physiological functions. These range from oxygen transport by hemoglobin to catalytic activities by various iron-containing enzymes. Requirement of iron for life can also be illustrated by the ability of the immune system to restrict microbial invasion by iron-sequestrating strategies (Ganz and Nemeth, 2015). Since there is no physiologically specialized excretory pathway for iron, the total body iron content and its distribution is tightly regulated by elaborate homeostatic mechanisms acting upon iron acquisition via the gastrointestinal tract and iron recycling by the reticuloendothelial system (Crielaard et al., 2017). Primary and secondary hemochromatoses are diseases of systemic iron mishandling, which culminate in iron overload and dysfunction of iron-laden organs (Fleming and Ponka, 2012). Worldwide impact of this condition can be exemplified by complications of thalassemia, one of the most common monogenic disorders with the reported incidence exceeding 68,000 cases/year (Weatherall, 2010). The increased iron burden in thalassemic patients is the result of uncontrolled dietary iron uptake and, in severe cases, repeated blood transfusion (Cao and Galanello, 2010). Excess iron subsequently accumulates in several vital organs, with the heart being one of the major targets (Siri-Angkul et al., 2018a). Unsurprisingly, a substantial fraction (> 60%) of thalassemia-related mortality is caused by cardiac complications (Borgna-Pignatti et al., 2005).

Cardiac iron deposition is markedly heterogenous and preferentially occurs in the basal segments of the anterior and inferior ventricular walls (Buja and Roberts, 1971; Walker, 2002). Patchy myocardial fibrosis has also been reported (Buja and Roberts, 1971), but details regarding iron overload-induced structural changes are lacking. Moreover, factors other than iron toxicity also contribute to cardiac remodeling (e.g., high-output hemodynamic status in iron-overload patients with inadequate hemoglobin). Thus, the exact role of iron in cardiac structural remodeling remains to be clarified. Both iron-overload condition and heart failure can contribute to the arrhythmia risk (Kremastinos and Farmakis, 2011). The dilated and remodeled cardiac chambers become arrhythmogenic substrate. However, arrhythmia incidence in patients with transfusion-dependent thalassemia was reportedly higher than the transfusion-independent form, even though the latter group had more severe volume overload and larger ventricular volumes (Aessopos et al., 2004). This clinical finding defies simple causal relationship between heart failure and arrhythmias and suggests the independent arrhythmogenic effect of iron toxicity.

Iron-overloaded hearts exhibits gradual deterioration in both mechanical function and electrical activity. Early detectable arrhythmias include occasional premature atrial/ventricular contractions and first-degree atrioventricular (AV) block, whereas sinoatrial node dysfunction, atrial flutter/fibrillation, second- and third-degree AV block, and ventricular tachycardia/fibrillation tend to occur in advanced stages of the disease (Engle et al., 1964; Kaye and Owen, 1978; Rosenqvist and Hultcrantz, 1989; Wang et al., 1994). Repolarization abnormality including nonspecific ST-T wave changes and QTc dispersion have also been reported (Ulger et al., 2006; Russo et al., 2011; Detterich et al., 2012). Despite these well-documented abnormal electrocardiographic findings, the fundamental aspect of iron-induced arrhythmogenesis is still incompletely understood. To encourage and facilitate further research into the elusive cellular mechanisms of arrhythmias in the siderotic heart, information from electrophysiological studies is summarized and discussed in this article. For detailed clinical information, please see a clinical review by Krematinos and Farmakis (Kremastinos and Farmakis, 2011).

## Effects of Iron Overload on Cardiac Action Potential Morphology

The first reported recording of action potential (AP) in iron-overloaded rat LV cardiomyocytes (Supplementary Table 1) demonstrated that 24-h incubation in ferric ammonium citrate (40–80 μg Fe/ml) caused reduced AP amplitude (APA), but the AP duration measured at 40% and 80% repolarization (APD_40_ and APD_80_) remained unchanged (Link et al., 1989). However, it has been shown later, also in rat LV cardiomyocytes, that incubation in the same concentrations of ferric ammonium citrate but with longer incubation time (72 h) was sufficient to induce shortened APD_50_ in addition to the reduced APA (Kuryshev et al., 1999). Consistently, LV cardiomyocytes isolated from Mongolian gerbils which had been subjected to chronic subcutaneous iron injection also had decreased APA and shortened APD_50_ (Kuryshev et al., 1999). In mouse SAN cardiomyocytes, chronic iron overload caused reduced APA, shortened APD_50_ and APD_90_, and a decreased spontaneous AP firing rate due to a decreased slope of diastolic depolarization (phase 4); however, the maximum diastolic potential and the slope of rapid depolarization (phase 0) were unaffected (Rose et al., 2011). Investigations into the effect of iron overload on the resting membrane potential (RMP) of ventricular cardiomyocytes in different models yielded different results. Slightly depolarized RMP (≈3–4 mV) has been observed in the LV cardiomyocytes from rats subjected to acute iron loading (80 μg Fe/ml, 24 h) (Link et al., 1989) and from Mongolian gerbils with chronic iron overload (Kuryshev et al., 1999). However, in the study by Kuryshev et al., this subtle RMP depolarization did not occur in iron-overloaded rat LV cardiomyocytes (40–80 μg Fe/ml, 72 h) (Kuryshev et al., 1999). Interspecies variation and different durations of iron exposure may account for these discordant findings.

Since the generation of reactive oxygen species (ROS) is likely to be a core pathophysiological process of iron-related cellular/organellar injury (Papanikolaou and Pantopoulos, 2005), research focusing on alterations of AP in the presence of oxidative stress should also be considered. Sequential three-phase changes of AP due to severe oxidative stress have been described in isolated rat and guinea-pig ventricular cardiomyocytes, specifically: (1) APD prolongation with early afterdepolarizations (EADs) and delayed afterdepolarizations (DADs), (2) shortened APD and depolarized RMP, and (3) overt and persistent depolarization leading to loss of excitability (Beresewicz and Horackova, 1991). Although the oxidative stress examined in this study was induced by direct treatment with hydrogen peroxide (H_2_O_2_), the results may be considered to represent the downstream effects of iron-induced ROS-mediated cellular damage on the electrical properties of the cardiomyocyte. Interestingly, ROS-induced afterdepolarizations have been mechanistically linked to overactivation of Ca^2+^/calmodulin kinase (CaMK) II in rabbit ventricular cardiomyocytes (Xie et al., 2009). As expected, treatment with deferoxamine could attenuate the H_2_O_2_-inducedAP changes (Beresewicz and Horackova, 1991), most likely by chelating the pre-existing intracellular iron, which could catalyze the conversion of H_2_O_2_ and superoxide anion (^•^O_2_^-^) to an even more potent ROS, hydroxyl radical (^•^OH) (Papanikolaou and Pantopoulos, 2005).

The precise mechanism for QTc prolongation/dispersion in patients with iron overload remains obscure. Since iron loading was shown to induce APD shortening in various models (Supplementary Table 1), current cellular experimental results seem to contradict the clinical findings. However, as mentioned earlier, ROS, which is the downstream product of iron-catalyzed redox reactions, could prolong the APD and induce afterdepolarizations/triggered activities (Beresewicz and Horackova, 1991; Xie et al., 2009). In addition, given the heterogeneity of cardiac iron deposition and fibrosis (Buja and Roberts, 1971; Walker, 2002), various degrees of repolarization abnormality can occur in different myocardial regions. These could contribute to QTc prolongation/dispersion.

## Effects of Iron Overload on Cardiac Calcium Channel Currents and Transporters

Altered AP parameters are the manifestation of the underlying changes in multiple trans-sarcolemmal ionic currents including those from Ca^2+^ channels. Under iron-overload conditions, voltage gated L-type and T-type Ca^2+^ channels (LTCC and TTCC) have been proposed as candidate molecular entrances into the cardiomyocyte for the ferrous form (Fe^2+^) of non-transferrin bound iron (NTBI) (Tsushima et al., 1999; Oudit et al., 2003; Kumfu et al., 2011; Lopin et al., 2012). This postulation has been supported by the fact that cardiac Fe^2+^ loading relies on electrical excitability of the heart and is sensitive to modulators of LTCC and TTCC (Tsushima et al., 1999; Oudit et al., 2003; Kumfu et al., 2011). However, the relative contribution of Ca^2+^ channels in iron uptake into the cardiomyocyte remains to be evaluated, given only a limited iron current via LTCC was observed even under a very high concentration of iron (15 mM) (Tsushima et al.,1999). Although the proof-of-concept experiment showing permeation and blockade of the TTCC by Fe^2+^in Ca_V_3.1-transfected HEK293 cells has been reported (Lopin et al., 2012; Supplementary Table 2), knowledge regarding the direct electrophysiological effects of iron overload on the natively expressed cardiac TTCC is still lacking. There are only a limited number of studies concerning altered LTCC function in iron-overloaded cardiomyocytes (Supplementary Table 2).

In isolated rat LV cardiomyocytes superfused with 2 mM Ca^2+^ plus various concentrations of iron, Fe^2+^, but not Fe^3+^, reversibly altered kinetic properties of the LTCC. Fe^2+^ (0.5–4 mM) delayed L-type Ca^2+^ current (*I*_Ca,L_) inactivation in a dose-dependent manner (Tsushima et al., 1999), possibly due to competitive binding of Fe^2+^ at the C-terminal Ca^2+^ binding site of the LTCC, thus attenuating Ca^2+^-mediated inactivation (Imredy and Yue, 1994; de Leon et al., 1995). Lower concentration of extracellular Fe^2+^ (0.5 mM) accentuated peak *I*_Ca,L_ and increased net Ca^2+^ influx via the LTCC, but these parameters were suppressed at higher Fe^2+^ concentrations (1–4 mM) (Tsushima et al., 1999). Even though the reduced peak *I*_Ca,L_ at high Fe^2+^ concentrations could potentially be explained by competitive channel permeation between Fe^2+^ and Ca^2+^, the mechanism by which the lower concentration of Fe^2+^ increased peak *I*_Ca,L_ remains obscure. It is also not known whether or not, and how, these iron-induced perturbations of the *I*_Ca,L_ affect the T-type Ca^2+^ current (*I*_Ca,T_). Hypothetically, in the face of impaired *I*_Ca,L_, cellular electrophysiological remodeling that leads to the compensatory upregulation of *I*_Ca,T_ channel may occur. Proving this hypothesis will clarify the relative roles of LTCC and TTCC in cardiac iron-overload conditions, as well as the role of pharmacological modulations of TTCC.

In contrast, LV cardiomyocytes from chronically iron-overloaded mice exhibited no changes in the peak, inactivation, and current-voltage relationship of *I*_Ca,L_ (Oudit et al., 2003). Different means of iron loading and the absence of Fe^2+^ in the superfusing extracellular solution during the patch-clamp recording in this study could be responsible for this discrepancy. Single-channel recording, which can provide additional information of single-channel conductance and gating property, has not been reported in both studies.

Effects of iron overload on *I*_Ca,L_ in other locations in the heart (besides the ventricles) have also been evaluated (Supplementary Table 2). In mice, a significant difference between the heart rates of control and chronically iron-treated groups could not be eliminated by complete sympathovagal blockade, suggesting that marked bradycardia in iron-overloaded animals was due to abnormal impulse generation by the SAN instead of autonomic dysfunction (Rose et al., 2011). Further experiments in isolated mouse SAN and right atrial appendage (RAA) cardiomyocytes revealed that peak *I*_Ca,L_ was reduced and a depolarizing shift of the current-voltage relationship was observed (Rose et al., 2011). It has been proposed that the preferential reduction of *I*_Ca,L_ at negative membrane potentials was the result of differential effects of chronic iron overload on two components of *I*_Ca,L_ expressed in the atria and the conducting system, Ca_V_1.2- and Ca_V_1.3-mediated I_Ca,L_ (Mangoni et al., 2003). Ca_V_1.3-mediated *I*_Ca,L_ is normally activated at a more negative membrane potential (≈-50 mV) compared to Ca_V_1.2-mediated *I*_Ca,L_ (≈-30 mV), and the depolarizing shift of *I*_Ca,L_ current-voltage relationship in iron-overloaded SAN and RAA cardiomyocytes resembled the results described in Ca_V_1.3-deficient mice (Zhang et al., 2002, 2005). Furthermore, *I*_Ca,L_ in LV cardiomyocytes, which does not express the Ca_V_1.3-dependent component, has been shown to be unaffected by chronic iron overload (Oudit et al., 2003). Thus, selective suppression of Ca_V_1.3-mediated *I*_Ca,L_ by chronic iron overload was suggested. This assumption has been supported by the finding that mRNA expression of Ca_V_1.3, but not Ca_V_1.2, was markedly reduced (> 40%) in RAA cardiomyocytes from chronically iron-overloaded mice. The attempt to measure the protein levels failed due to a technical error (Rose et al., 2011).

Abnormal trans-sarcolemmal and subcellular Ca^2+^ fluxes mediated by various transport mechanisms, other than the above-discussed Ca^2+^ currents, may contribute to electrical instability of the cardiomyocyte (Ter Keurs and Boyden, 2007; Zhao et al., 2012). These include the potential involvement of the Ca^2+^ handling processes in iron-induced arrhythmogenesis. Growing evidence indicates that oxidative stress accompanying iron-overload conditions can interfere with the function of multiple Ca^2+^cycling machineries (Khamseekaew et al., 2016; Gordan et al., 2018). Excess ROS could impair cytosolic Ca^2+^ removal by the Na^+^-Ca^2+^ exchanger (NCX) (Xu et al., 1997), sarcolemmal Ca^2+^ ATPase (Kaneko et al., 1989a,b), and sarco/endoplasmic reticulum Ca^2+^ ATPase (SERCA) (Zeitz et al., 2002). Moreover, Fe^2+^exerts its ROS-independent inhibitory effect on ryanodine receptors (RyR) via competitive binding, thus compromising Ca^2+^-induced Ca^2+^ release from the sarcoplasmic reticulum (Kim et al., 1995). The causal relationship between this complex spatiotemporal Ca^2+^ dysregulation and membrane potential fluctuation (EADs, DADs, and triggered activities) is yet to be systematically proven in iron-overloaded cardiomyocytes.

## Effects of Iron Overload on Cardiac Sodium Channel Currents

Consistent with the decreased AP amplitude, patch clamp recording demonstrated dose- and time-dependent reduction of peak Na^+^ current (*I*_Na_) in short-term iron-incubated (80 μg Fe/ml, 72 h) cultured rat LV cardiomyocytes and also in freshly isolated LV cardiomyocytes from chronically iron-injected gerbils (Kuryshev et al., 1999). Iron overload shifted the inactivation curve of *I*_Na_ toward negative voltages and delayed the recovery from inactivation in both species (Kuryshev et al., 1999). Single-channel recording revealed that Na^+^ channels from the iron-incubated rat LV cardiomyocytes had reduced opening probability and a decreased average single-channel current (Kuryshev et al., 1999). The amount of Na^+^ channel protein was unaffected (Kuryshev et al., 1999). These findings are summarized in Supplementary Table 2.

## Effects of Iron Overload on Cardiac Potassium Channel Currents

Multiple K^+^ currents play important roles in the repolarization phase of AP and are the key determinant of the resting membrane potential. Several members of this heterogeneous group were shown to be affected by iron overload conditions (Supplementary Table 2). In rats and gerbils LV cardiomyocytes subjected to acute and chronic iron loading protocols, the peak of transient outward K^+^ current (*I*_to_) was increased, and a small depolarizing shift (≈5 mV) of its inactivation curve was observed (Kuryshev et al., 1999). However, the inward rectifier K^+^ current (*I*_K1_) was unaffected (Kuryshev et al., 1999).

In guinea-pig LV cardiomyocytes, the adenosine triphosphate (ATP)-sensitive K^+^(K_ATP_) channel current (*I*_KATP_) could be activated by iron-induced oxidative stress (Tokube et al., 1998). Interestingly, activating the cardiac K_ATP_ channel could increase ^•^OH levels, whereas blocking it led to decreased ^•^OH levels in rat myocardium (Obata and Yamanaka, 2000; Han et al., 2002). These findings indicate the presence of reciprocal positive-feedback interactions between ROS production and the altered function of the K_ATP_ channel. Moreover, the inhibitory effect of ROS on the human ether-a-go-go-related gene (hERG) K^+^ channels, which mediate the rapid delayed rectifier K^+^ current (*I*_Kr_) in the heart, has already been studied in various cell types (Vandenberg, 2010). Nevertheless, the specific effects of iron overload on cardiac *I*_Kr_ as well as the link to cardiac repolarization abnormality have not been clearly established. Changes in *I*_Kr_ and/or *I*_Ks_ may eventually lead to altered maximum diastolic potential. In this way, indirect chronotropic effect may ensue. Thus, the relationships between various K^+^ currents and iron-derived ROS highlight another interesting domain to be explored.

## Effects of Iron Overload on Hyperpolarization-Activated Pacemaker Currents

Sinus bradycardia and SAN dysfunction have been observed in iron overload patients (Detterich et al., 2012), and in chronically iron-overloaded mice (Rose et al., 2011). SAN cardiomyocytes from mice subjected to chronic iron overload exhibited decreased slope diastolic depolarization (Rose et al., 2011). Although the hyperpolarization-activated pacemaker current (*I*_h_ or *I*_f_) is a major contributor of spontaneous depolarization in SAN cells, Rose et al. reported no significant changes in *I*_f_ density and current-voltage relationship in mice with chronic iron overload (Rose et al., 2011; Supplementary Table 2). Therefore, decreased Ca_V_1.3-mediated *I*_Ca,L_ has been proposed as the underlying mechanism of the suppressed SAN automaticity (Rose et al., 2011).

## Forging the Field: Additional Issues to Be Explored in Iron-Associated Cardiac Pathoelectrophysiology

In iron-overload patients, cardiac ion channels operate in the presence of both high intracellular labile iron pool and high extracellular NTBI. Most cellular electrophysiological studies exploited chronic iron loading in various species, while in some studies the cardiomyocytes were briefly incubated in iron or acutely exposed to the iron-containing solution at the time of patch-clamp recording (Supplementary Tables 1, 2). Thus, different iron-loading protocols, especially in terms of the duration of iron exposure, may lead to discrepant findings, as seen in the case of *I*_Ca_-suppressing effects in acute and chronic iron overload models (Tsushima et al., 1999; Oudit et al., 2003). Moreover, other pathways for iron uptake into the cardiomyocyte exist, including receptor-mediated endocytosis of the well-recognized transferrin-Fe^3+^ complex and a recently described lipocalin 2-siderophore-Fe^3+^ complex (Yang et al., 2002; Xu et al., 2012; Siri-Angkul et al., 2018b). It remains to be examined whether these forms of cardiac iron-loading, bypassing the ion channel-mediated transport system, indirectly exert any effects on the electrophysiological properties of cardiomyocytes.

Additionally, investigating deeper into the genomic and molecular mechanisms (i.e., abnormal gene expression, aberrations in posttranslational modifications or trafficking of ion channels and their regulatory proteins, and interactions between the electrophysiological events and other biochemical processes) is of no less importance. This point can be exemplified by the complexity and multifaceted nature of intracellular Ca^2+^ regulation. Understanding of how deranged Ca^2+^ cycling influences the membrane excitability, in the specific context of iron-overloaded cardiomyocyte, is still incomplete.

The main purpose of this article is to provide the molecular/cellular basis of iron-induced cardiac pathoelectrophysiology. However, it should be noted that additional data from other aspects are also essential for bridging the gap of knowledge between the cellular phenomena and the clinical phenotypes. These include, but not limited to, alterations in intercellular coupling, roles of non-parenchymal cells (e.g., fibroblasts and macrophages), structural remodeling, systemic immunoinflammatory responses, and coincident extracardiac pathology (e.g., iron-induced endocrinopathies and autonomic disturbances) (Kremastinos and Farmakis, 2011). Due to lack of information, the extent and the characteristics of interactions between these phenomena and cellular electrophysiological changes are difficult to be determined at this time.

Despite incomplete characterization of iron-induced K^+^ current alterations, amiodarone has been used to treat supraventricular arrhythmias commonly found in iron-overload patients (Pennell et al., 2013). Potential role of LTCC blockers as an inhibitor of cardiac iron uptake is also being evaluated in clinical trials (Fernandes et al., 2013, 2016; Shakoor et al., 2014; Eghbali et al., 2017; Sadaf et al., 2018). It is still unknown whether the use of LTCC blockers will exert any significant electrophysiological effects. Clinically, diseases of the cardiac rhythm can be classified according to their ECG features and/or functional anatomical characteristics (e.g., atrial arrhythmias, ventricular arrhythmias, etc). However, for siderotic hearts, current data are still insufficient to formulate specific pathophysiological explanations for such clinical entities. We hope that, with more information in the future, iron overload-induced arrhythmias could be revisited with this useful functional anatomy-based approach.

## Conclusion

Although some perturbations of ionic channels/currents of iron-induced electrical disturbances in the heart have already been characterized, current electrophysiological knowledge regarding the siderotic heart is still far from complete, especially the lack of data in human cardiomyocytes. Further cellular electrophysiological insights are certain to provide a firm base for our in-depth understanding and for future investigations in more complicated systems (e.g., myocardial tissue, whole-heart, and clinical cardiac electrophysiology), with identification of potential therapeutic targets and development of practical treatment strategies being the extremely worthwhile goals.

## Author Contributions

NS-A, L-HX searched the literature, collected the data, analyzed the data, and wrote the manuscript. SC, NC studied the design, searched the literature, analyzed the data, and wrote the manuscript.

## Conflict of Interest Statement

The authors declare that the research was conducted in the absence of any commercial or financial relationships that could be construed as a potential conflict of interest.
