# Supplementary material for: Cellular Electrophysiology of Iron-Overloaded Cardiomyocytes
Source: Front Physiol. 2018 Nov 15;9:1615. doi: 10.3389/fphys.2018.01615 (PMC6249272; doi:10.3389/fphys.2018.01615)
Supplement: Supplementary file 1 [file Table_1.docx]

**Table 1. Effects of iron on cardiac action potential (AP) morphology**

| **Model** | **Iron-loading protocol** | **Iron exposure during patch-clamp recording** | **RMP** | **Changes of AP morphology** | | | | **Interpretation** |
| --- | --- | --- | --- | --- | --- | --- | --- | --- |
|  |  |  |  | **APA** | **APD** | **Phase 0 slope** | **Phase 4 slope** |  |
| Cultured neonatal rat LV cardiomyocytes (Link et al., 1989) | Incubation in ferric ammonium citrate 20, 40 or 80 µg Fe/ml for 24 h | No | Depolarized (≈3 mV) in 80 µg Fe/ml | ↓ | ↔ | ↔ | N/A | Acute iron overload caused slight RMP depolarization and reduced APA in LV cardiomyocytes. |
| Cultured neonatal rat LV cardiomyocytes (Kuryshev et al., 1999) | Incubation in ferric ammonium citrate 40 or 80 µg Fe/ml for 24-72 h | No | ↔ | ↓ | ↓ | N/A | N/A | Acute iron overload caused APA reduction and APD shortening in LV cardiomyocytes. |
| Mongolian gerbil (Kuryshev et al., 1999) | Iron dextran 200 mg Fe/kg/wk SC for 8 wks prior to epicardial LV cardiomyocyte isolation | No | Depolarized (≈4 mV) | ↓ | ↓ | N/A | N/A | Chronic iron overload caused APA reduction, APD shortening, and slightly depolarized RMP in LV cardiomyocytes. |
| CD1 mice (Rose et al., 2011) | Iron dextran 600 mg Fe/kg IP 3 days/wk for 4 wks prior to cardiomyocyte isolation from SAN | No | ↔  (maximum diastolic potential) | ↓ | ↓ | ↔ | ↓ | Chronic iron overload caused APA reduction, APD shortening, and reduced firing rate due to decreased phase 4 slope in SAN cardiomyocytes. |

AP, action potential; APA, action potential amplitude; APD, action potential duration; IP, intraperitoneal injection; LV, left ventricle; RMP, resting membrane potential; SAN, sinoatrial node; SC, subcutaneous injection
