# Supplementary material for: Cellular Electrophysiology of Iron-Overloaded Cardiomyocytes
Source: Front Physiol. 2018 Nov 15;9:1615. doi: 10.3389/fphys.2018.01615 (PMC6249272; doi:10.3389/fphys.2018.01615)
Supplement: Supplementary file 2 [file Table_2.docx]

**Table 2. Effects of iron on cardiac ion channel functions and trans-sarcolemmal ionic currents**

| **Model** | **Iron-loading protocol** | **Iron exposure during patch-clamp recording** | **Changes in ion channels/ionic currents** | | | | **Interpretation** |
| --- | --- | --- | --- | --- | --- | --- | --- |
|  |  |  | **Peak current density** | **Current-voltage**  **relationship** | **Inactivation** | **Other major findings** |  |
| **L-type Ca^2+^ channel and current** | | | | | | | |
| Isolated rat LV cardiomyocytes (Tsushima et al., 1999) | Superfusion with 0.25-4 mM Fe^2+^ | Yes | ↔ In 0.25 mM  ↑ In 0.5 mM  ↓ In >0.5 mM  (reversible) | No shift (↓ peak only) | Delayed in ≥0.5mM  (reversible) | Altered *I*_Ca,L_ time integral (corresponded to changes in peak *I*_Ca,L_) | ● Low Fe^2+^concentration potentiated *I*_Ca,L_.  ● High Fe^2+^ concentration attenuated *I*_Ca,L_.  ● Fe^2+^caused dose-dependent delay of *I*_Ca,L_ inactivation. |
| B6D2F1 mice (Oudit et al., 2003) | Iron dextran 400 mg Fe/kg IP 5 days/wk for 4 wks prior to cardiomyocyte isolation from LV | No | ↔ | ↔ | ↔ | N/A | Chronic iron overload did not alter *I*_Ca,L_ in LV cardiomyocytes. |
| CD1 mice (Rose et al., 2011) | Iron dextran 600 mg Fe/kg IP 3 days/wk for 4 wks prior to cardiomyocyte isolation from SAN and RAA | No | ↓ | ↓ Peak with depolarizing shift | N/A | ↓ mRNA of Ca_V_1.3, but not Ca_V_1.2 | Chronic iron overload preferentially reduced  Ca_V_1.3-mediated *I*_Ca,L_ in SAN and atria. |
| **T-type Ca^2+^ channel and current** | | | | | | | |
| T-type Ca^2+^ channel (Ca_V_3.1)-transfected HEK293 cells (Lopin et al., 2012) | Superfusion with 0.25-1.85 mM Fe^2+^ for the study of *I*_Ca,T_ alterations and 1-9 mM Fe^2+^ for the study of Fe^2+^ permeation through the T-type Ca^2+^ channel | Yes | ↓ | ↓ Peak with depolarizing shift | ↔ | Small inward current could be detected when all other extracellular cations were replaced by 1-9 mM Fe^2+^ | Fe^2+^ reduced *I*_Ca,T_ and could permeate through the T-type Ca^2+^ channel |
| **Na^+^ channel and current** | | | | | | | |
| Cultured neonatal rat LV cardiomyocytes (Kuryshev et al., 1999) | Incubation in ferric ammonium citrate 40 or 80 µg Fe/ml for 24-72 h | No | ↓ | No shift (↓ peak only) | Hyper-polarizing shift of inactivation curve with delayed recovery | ● ↓ Na^+^ channel opening probability  ● ↔ Na^+^ channel protein level | Acute iron overload reduced *I*_Na_ and delayed its recovery from inactivation by altering the gating property, but not protein level, of Na^+^ channels in LV cardiomyocytes. |
| Mongolian gerbil (Kuryshev et al., 1999) | Iron dextran 200 mg Fe/kg/wk SC for 8 wks prior to epicardial LV cardiomyocyte isolation | No | ↓ | No shift (↓ peak only) | Hyper-polarizing shift of inactivation curve with delayed recovery | N/A | Chronic iron overload reduced *I*_Na_ and delayed its recovery from inactivation in LV cardiomyocytes. |
| **K^+^ channels and currents** | | | | | | | |
| Cultured neonatal rat LV cardiomyocytes (Kuryshev et al., 1999) | Incubation in ferric ammonium citrate 40 or 80 µg Fe/ml for 24-72 h | No | ↑ *I*_to_  ↔ I_K1_ | No shift (↑*I*_to_ only) | Depolarizing shift of *I*_to_ inactivation curve | N/A | Acute iron overload accentuated *I*_to_, but not *I*_K1_, in LV cardiomyocytes |
| Mongolian gerbil (Kuryshev et al., 1999) | Iron dextran 200 mg Fe/kg/wk SC for 8 wks prior to epicardial LV cardiomyocyte isolation | No | ↑ *I*_to_  ↔ *I*_K1_ | No shift (↑*I*_to_ only) | Depolarizing shift of *I*_to_ inactivation curve | N/A | Chronic iron overload accentuated *I*_to_, but not *I*_K1_, in LV cardiomyocytes |
| Isolated guinea- pig LV cardiomyocytes  (Tokube et al., 1998) | Acute treatment with XO (0.1 U/ml) + HX (0.5 µM) + Fe^3+^ (0.1 mM) to generate ROS | Yes | ↑ *I*_KATP_; adding mannitol (ROS scavenger) attenuated the increase | N/A | N/A | ↑ K_ATP_ channel opening probability | Iron-induced oxidative stress activated K_ATP_ channel in LV cardiomyocytes |
| **Hyperpolarization-activated pacemaker current** | | | | | | | |
| CD1 mice (Rose et al., 2011) | Iron dextran 600 mg Fe/kg IP 3 days/wk for 4 wks prior to cardiomyocyte isolation from SAN | No | ↔ | ↔ | N/A | N/A | Chronic iron overload did not alter *I*_f_ in SAN cardiomyocytes. |

*I*_Ca,L_, L-type calcium current; *I*_Ca,T_, T-type calcium current; *I*_f_, hyperpolarization-activated pacemaker current; *I*_K1_, inward rectifier potassium current; *I*_KATP_, ATP-sensitive potassium current; *I*_Na_, sodium current; *I*_to_, transient outward potassium current; IP, intraperitoneal injection; LV, left ventricle; RAA, right atrial appendage; ROS, reactive oxygen species; SAN, sinoatrial node; SC, subcutaneous injection; XO, xanthine oxidase; HX, hypoxanthine oxidase
